# mCRPC progression of disease after [^177^Lu]Lu-PSMA-617 detected on [^18^F]Choline: a case of PCa heterogeneity

**DOI:** 10.1016/j.eucr.2024.102750

**Published:** 2024-05-08

**Authors:** Riccardo Laudicella, Fabio Minutoli, Simona Russo, Massimiliano Siracusa, Michelangelo Bambaci, Benedetta Pagano, Sergio Baldari

**Affiliations:** a Nuclear Medicine Unit, Department of Biomedical and Dental Sciences and of Morpho-Functional Imaging, University of Messina, 98121, Italy; b Nuclear Medicine Unit, Humanitas Istituto Clinico Catanese, Misterbianco, 95045, Italy

**Keywords:** FDG, Metastatic castration-resistant prostate cancer, PRRT, RLT, Theranostics

## Abstract

Radioligand therapy with [^177^Lu]Lu-PSMA is a theranostic approach for heavily treated mCRPC patients with positive PSMA PET in the absence of relevant PSMA-negative metastases assessed through CT, MRI, bone scan or FDG PET. In this case, we described a mCRPC patient treated with RLT with discordant PSA values and PSMA images, in which Choline PET confirmed a biochemically suspected disease progression (PD), showing metastatic lesions not revealed by PSMA imaging.

## 1 Introduction

[^177^Lu]Lu-PSMA radioligand therapy (RLT) is a theranostic opportunity for patients with prostate-specific membrane antigen (PSMA)-positive metastatic castration-resistant prostate cancer (mCRPC) after androgen receptor pathway inhibitor (ARPI) and taxane-based chemotherapy.^1^, ^2^, ^3^ Before RLT and according to guidelines^3^, mCRPC patients should undergo a PSMA-positron emission tomography (PET) demonstrating the presence of PSMA-avid disease (tumor uptake ≥0.5-fold of parotid), coupled with computed tomography (CT), or magnetic resonance imaging, or bone scan or (in selected cases) fluorodeoxyglucose (FDG) PET, to rule out relevant PSMA-negative metastases or active/lytic bone lesions. Choline PET is a molecular imaging technique that, despite a lower accuracy than PSMA PET (especially for PSA <1 ng/ml), still represents a valuable diagnostic tool assessing the phospolipidic metabolism usually increased in advanced PCa. Here, we describe the case of a mCRPC patient treated with RLT in which Choline PET confirmed a biochemically suspected disease progression (PD), showing metastatic lesions not revealed by PSMA imaging.

## 2 Case presentation

**Fig. 1:**
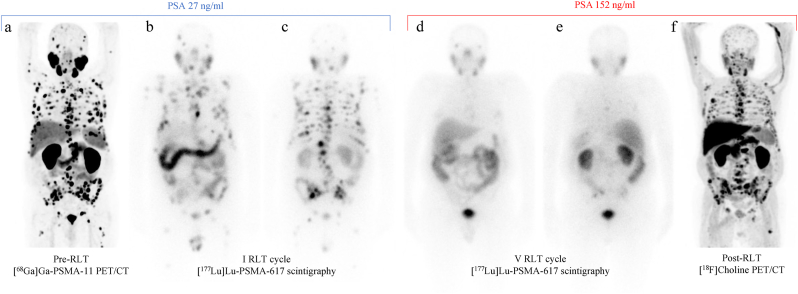
**a)** Pre-RLT maximum intensity projection (MIP) [^68^Ga]Ga-PSMA-11 PET/CT showing intense uptake in many bone lesions; **b)** anterior and **c)** posterior planar [^177^Lu]Lu-PSMA-617 biodistribution scintigraphy after the I cycle, confirming high PSMA-uptake in the known bone lesions; **d)** anterior and **e)** posterior planar [^177^Lu]Lu-PSMA-617 biodistribution scintigraphy after the V cycle showing an evident reduction in number and PSMA-uptake of the known bone lesions; **f)** post-RLT MIP [^18^F]Choline PET/CT showing many bone lesions with variable uptake.

**Fig. 2:**
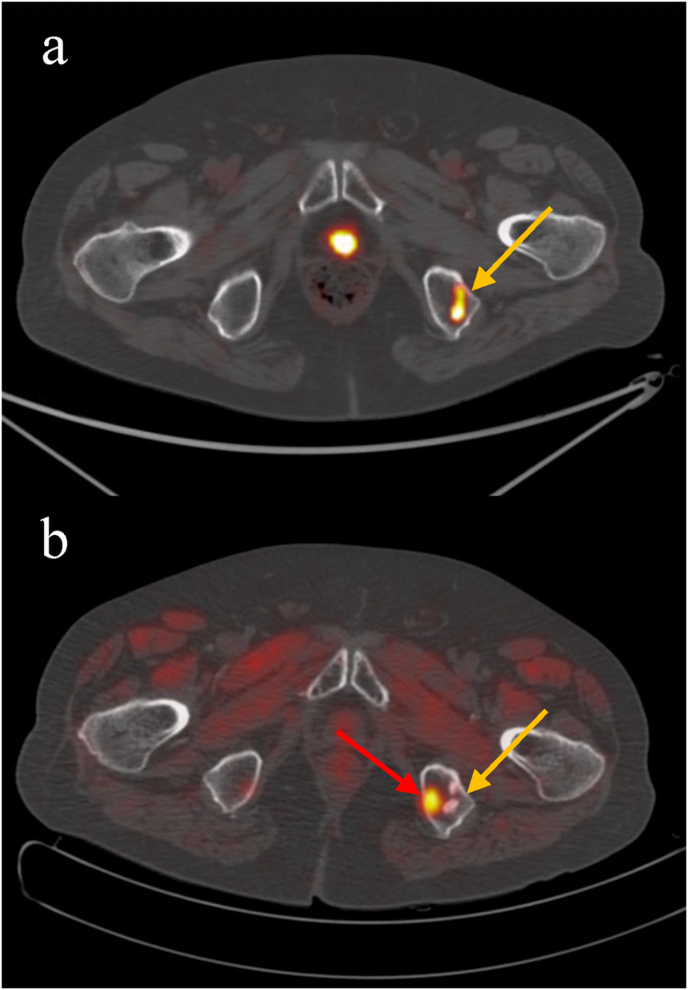
**a)** Pre-RLT axial fused [^68^Ga]Ga-PSMA-11 PET/CT showing intense uptake in sclerotic lesions of the left ischiatic bone (orange arrow); **b)** post-RLT axial fused [^18^F]Choline PET/CT showing faint uptake in the known sclerotic lesions of the left ischiatic bone (orange arrow) associated with the presence of a focal and intense medullary localizations (red arrow). (For interpretation of the references to colour in this figure legend, the reader is referred to the Web version of this article.)

A 77-year-old patient with International Society of Urological Pathology (ISUP) grade group 2 mCRPC heavily treated (radical prostatectomy, local radiation therapy, ARPIs, androgen deprivation therapy, and chemotherapies) with no breast cancer (BRCA) gene expression, was considered for PSMA RLT. Therefore, the patient underwent a [^68^Ga]Ga-PSMA-11 PET/CT showing many PSMA-positive sclerotic bone metastases (Fig. 1a), and a bone scan showing no additional lesions. With a prostate-specific antigen (PSA) of 27 ng/ml, a normal alkaline phosphatase (ALP) of 120 U/L and the presence of mild pain, the patient started the RLT at a fixed dose of 7.4 GBq per cycle. At [^177^Lu]Lu-PSMA-617 biodistribution scintigraphic and single-photon emission computed tomography (SPECT)/CT images performed within 48h after therapeutic administration, we registered a satisfactory, even if partial, response (Fig. 1b, c, 1d, 1e). However, despite a reduction of ALP (up to 67.5 U/L) and pain disappearance, we observed a continuous PSA increase of up to 152 ng/ml at the V cycle, suspicious for PD. The case was discussed in a multidisciplinary meeting, considering the best imaging option able to reveal the real disease burden. Due to the aforementioned reduction in PSMA expression on the last RLT biodistribution images (V cycle; Fig. 1d and e) and the absence of aggressive disease clinical signs, instead of a PSMA or FDG PET, the patient underwent [^18^F]Choline PET/CT (Fig. 1f) to quantify a highly suspicious PSMA-negative disease. Indeed, [^18^F]Choline PET/CT showed variable radiotracer uptake (absent to high) in the known PSMA-PET positive sclerotic bone metastases (Fig. 2a) coupled with the presence of several medullary localizations (Fig. 2b), not evident at the initial PSMA imaging (Fig. 2a). Such findings were indicative of PD, as confirmed by a further PSA increase up to 249 ng/ml one month thereafter.

## 3 Discussion

In our case, Choline PET revealed the presence of PSMA-negative lesions confirming a clinically suspected PD after RLT. This case further shed light on the complex heterogeneity of PCa^4^ and the eventuality of disease dedifferentiation (loss of PSMA expression) in the case of heavily treated patients.^5^ It is well known the role of the metabolic tracer FDG to reveal biologically aggressive disease in advanced PCa patients^1^^,^^3^, especially when PSMA imaging results and PSA values are discordant. Here we showed, for the first time in the literature, the opportunity offered by the metabolic tracer Choline in a case of advanced PCa patient treated with [^177^Lu]Lu-PSMA RLT to show the real disease burden. Such an approach can potentially improve patients’ selection for RLT highlighting unresponsive/dedifferentiated PSMA-non avid lesions.

## 4 Conclusion

[^18^F]Choline PET/CT may be a useful tool in [^177^Lu]Lu-PSMA RLT patients in case of discordant PSA values and PSMA imaging.

## Funding

The authors declare that no funds, grants, or other support were received during the preparation of this manuscript.

## Declaration of interest

None.

## Data availability

The data analysed during the current study are available from the corresponding author on reasonable request.

## Ethics approval

This observational study was performed in line with the principles of the Declaration of Helsinki.

## Informed consent

Informed consent was obtained from the individual participant included in the study.

## Consent to publish

The authors affirm that human research participants provided informed consent for the publication of the images in the Figure.

## CRediT authorship contribution statement

**Riccardo Laudicella:** Conceptualization, Data curation, Formal analysis, Writing – original draft. **Fabio Minutoli:** Supervision, Writing – review & editing. **Simona Russo:** Investigation. **Massimiliano Siracusa:** Investigation. **Michelangelo Bambaci:** Data curation. **Benedetta Pagano:** Resources. **Sergio Baldari:** Supervision.
